# Case Report: 3D-CISS and PC-MRI in the diagnosis and surgical planning of hydrocephalus secondary to presumptive lateral aperture obstruction in a dog

**DOI:** 10.3389/fvets.2025.1646137

**Published:** 2025-10-10

**Authors:** Anna Tauro, Manabu Kurihara, John Macri, Peter Early, Christopher L. Mariani, Natasha J. Olby, Karen R. Muñana, Melissa J. Lewis, Linda Sjalander-Dillenbeck

**Affiliations:** ^1^Department of Clinical Sciences, College of Veterinary Medicine, North Carolina State University, Raleigh, NC, United States; ^2^Neurology and Neurosurgery Service, Access Specialty Animal Hospital, Pasadena, CA, United States; ^3^Molecular Biomedical Sciences, College of Veterinary Medicine, North Carolina State University, Raleigh, NC, United States; ^4^Veterinary Radiology, Department of Environmental Health and Radiological Sciences, Colorado State University, Fort Collins, CO, United States

**Keywords:** tetraventricular hydrocephalus, ventricle-to-brain index, syringomyelia, ventriculo-peritoneal shunt, lateral aperture obstruction, PC-MRI, 3D-CISS, dog

## Abstract

**Introduction:**

This report describes the use of three-dimensional constructive interference in steady state (3D-CISS) and phase-contrast magnetic resonance imaging (PC-MRI) sequences to investigate the etiology of severe hydrocephalus, and the subsequent surgical management and long-term outcome.

**Case presentation:**

A 5-month-old male Rhodesian Ridgeback presented with acute, progressive neurological signs culminating in non-ambulatory tetraparesis. Clinical and imaging findings were consistent with non-communicating tetraventricular hydrocephalus with concurrent severe syringomyelia, but conventional MRI failed to identify the cause of cerebrospinal fluid (CSF) flow obstruction. Following failure of medical management, advanced MRI sequences were performed to clarify the underlying etiology and guide surgical planning. These included 3D-CISS for high-resolution anatomical assessment and PC-MRI for dynamic evaluation of CSF flow. The findings demonstrated patent intraventricular CSF flow and no evidence of arachnoid septations within the fourth ventricle, thereby excluding a fourth ventricle arachnoid diverticulum. Instead, the imaging findings supported a presumptive diagnosis of hydrocephalus secondary to lateral aperture occlusion. A ventriculoperitoneal (VP) shunt was placed, resulting in substantial clinical improvement, although mild residual cerebellar deficits persisted. Five months later, the dog experienced acute deterioration. Computed tomography revealed fracture of the distal catheter at the level of its abdominal wall anchoring site, likely due to progressive tension as the dog grew, resulting in the catheter snapping into two segments. Revision surgery restored CSF diversion and led to rapid clinical recovery. The patient remains neurologically stable at 28 months of age, 15 months post-revision.

**Conclusion:**

Advanced MRI sequences, particularly 3D-CISS and PC-MRI, were instrumental in supporting the presumptive diagnosis of lateral aperture obstruction as the underlying cause of hydrocephalus and in guiding an individualized, effective surgical strategy. This case highlights the diagnostic and clinical value of advanced MRI techniques in managing complex hydrocephalus.

## 1 Introduction

Hydrocephalus is a neurological disorder characterized by an abnormal accumulation of cerebrospinal fluid (CSF) within the cranial cavity and it can be associated with increased intracranial pressure, which may contribute to progressive cerebral dysfunction (1–3). It is frequently described in young animals of small and toy breeds (1, 2), but the condition encompasses a spectrum of etiologies and should not be presumed to be congenital without thorough diagnostic evaluation.

Hydrocephalus can be classified according to the location of CSF accumulation, the underlying cause (congenital versus acquired), and CSF flow dynamics (1, 2). Based on location, *internal* hydrocephalus involves CSF accumulation within the ventricles (1), whereas *external* hydrocephalus, a rarer form, involves CSF accumulation predominantly in the subarachnoid space with only mild or absent ventricular dilation (1, 4). From a physiological standpoint, all forms of hydrocephalus involve some degree of CSF flow obstruction (2) and are further divided into *communicating* (extra-ventricular obstructive) and *non-communicating* (intra-ventricular obstructive) types (1, 2).

The current understanding of CSF dynamics favors a bidirectional and pulsatile flow model, primary driven by the cardiac cycle (5). Cerebrospinal fluid is predominantly produced by the choroid plexus in all four ventricles, with the fourth ventricle contributing the largest proportion in dogs—accounting for approximately 55% of the total choroid plexus surface area (5, 6). From the lateral ventricles, CSF flows through the interventricular foramina (foramina of Monro in people) into the third ventricle, then continues through the mesencephalic aqueduct to the fourth ventricle (5–9). In dogs, the fourth ventricle communicates with the subarachnoid space via paired lateral apertures, equivalent to the foramina of Luschka in people (5, 7, 8). The median aperture (foramen of Magendie) described in people is absent in dogs (10). CSF also flows caudally into the central canal of the spinal cord, reaching the terminal ventricle located within the conus medullaris (5, 8). CSF absorption primarily occurs via lymphatic pathways, including the perineural sheaths of cranial nerves—especially the olfactory nerves via the cribriform plate into nasal and cervical lymphatics (5). Additional efflux routes include the optic and trigeminal nerves, meningeal lymphatics, and to a lesser extent, arachnoid granulations, which may play a supplementary, pressure-dependent role (5). There is also emerging evidence that a small proportion of CSF may be reabsorbed by the choroid plexus itself (6). In addition to classical bulk flow routes, there is increasing recognition of CSF movement along perivascular spaces through the brain parenchyma—commonly referred to as the glymphatic system—which may contribute to waste clearance and interstitial fluid exchange, particularly during sleep and in a pressure-independent manner (5).

Hydrocephalus and syringomyelia—the latter characterized by fluid accumulation within the spinal cord—may share a common underlying mechanism involving disrupted CSF dynamics.

In this report, we use the term *syringomyelia* rather than *syringohydromyelia*. Syringomyelia refers to a glia-lined fluid-filled cavity within the spinal cord parenchyma that is separate from the central canal, whereas hydromyelia describes dilatation of the central canal lined entirely by ependyma. The term syringohydromyelia is sometimes used to describe a cavity that represents a dilated central canal partially lined by ependyma but also extending into the spinal cord parenchyma. However, post-mortem and experimental studies have demonstrated that even minor central canal dilatation results in disruption of the ependymal lining, and that all syringomyelic cavities ultimately communicate with the central canal at some level of the spinal cord. For this reason, and in line with the approach recommended by Rusbridge et al. (11), the simpler term syringomyelia is used throughout this report.

In hydrocephalus, obstruction of CSF outflow can raise intracranial pressure and alter pulsatile flow at the cranio-cervical junction, leading to abnormal pressure gradients (5, 11). These gradients may force CSF into the spinal cord parenchyma and contribute to syrinx formation (5, 11). As such, hydrocephalus may play a role in the pathogenesis of syringomyelia by disturbing normal CSF flow patterns (5, 11).

Common clinical signs of hydrocephalus include a dome-shaped calvarium, obtundation, behavioral changes, ataxia, seizures, ventrolateral strabismus, and central blindness (12, 13). Diagnosis typically relies on advanced imaging, with magnetic resonance imaging (MRI) and computed tomography (CT) being the most frequently employed techniques (12, 13). MRI is considered the modality of choice, offering better sensitivity than CT for detecting underlying causes and estimating intraventricular pressure through surrogate markers (1, 12, 14).

Medical therapy—including corticosteroids, omeprazole, furosemide, and acetazolamide—has been reported to provide only limited or temporary benefits (12, 15–22). Consequently, long-term management usually requires surgical intervention. Surgical options include removing the underlying obstruction when feasible, or more commonly, implanting a ventriculoperitoneal (VP) shunt (2). The primary surgical goal is to reduce intraventricular volume by diverting CSF from the lateral ventricles, typically via a VP shunt to the peritoneal cavity, or less commonly via a ventriculoatrial shunt to the right atrium of the heart (2, 23).

We report a case of a young, large-breed dog with severe tetraventricular hydrocephalus and syringomyelia, suspected to result from lateral aperture obstruction. This report highlights the role of advanced MRI techniques in differentiating this condition from other forms of non-communicating (intraventricular obstructive) hydrocephalus and provides details on the surgical intervention, associated complications, and long-term outcome.

## 2 Case description

A 5-month-old male Rhodesian Ridgeback was presented with a 10-day history of regurgitation, weight loss, right head tilt, and weakness progressing to non-ambulatory tetraparesis. On presentation, physical examination revealed moderate generalized muscle atrophy. Neurological examination revealed obtundation, a right head tilt, moderate scoliosis, non-ambulatory tetraparesis, reduced postural reactions in all four limbs, and diminished withdrawal (flexor) reflexes in the thoracic limbs (Table 1). Neurolocalization was multifocal, involving the brainstem and cervical spinal cord, particularly the C6–T2 segments.

**Table 1:** Chronological overview of the dog’s age, body weight, clinical history, imaging (CT/MRI), and surgical interventions, from initial presentation to the last follow-up at 28 months of age.

Age	BW (Kg)	Clinical history and signs	Imaging	Surgery
5 months	17	Initially presented recumbent. Brain MRI performed. Corticosteroid treatment initiated, leading to weak ambulation.	MRI	
		(12 days later) Mild improvement, but persistent mild tetraparesis and right head tilt.		
7 months	25	Neurological deterioration to non-ambulatory status. Corticosteroid dose increased.		
8 months		Neurologically unchanged.		
		(10 days later) Second MRI with advanced sequences performed, followed by VP shunt placement. Patient progressively improved and began ambulating independently one week after surgery.	Advanced MRI Post-op radiograph	VP shunting
11 months	N/A	Strongly ambulatory with mild hypermetria in the thoracic limbs.		
13 months	42	Neurological deterioration: non-ambulatory tetraparesis. CT scan revealed undershunting due to distal catheter fracture. Revision surgery performed. The patient regained ambulation, though with severe cerebellar tetra-ataxia.	CT pre-op Post-op radiograph	Revision surgery
20 months	N/A	Improving: mild cerebellar tetra-ataxia.		
24 months	N/A	Stable: mild cerebellar tetra-ataxia.	CT	
28 months	40	Stable: mild cerebellar tetra-ataxia.		

Hematology and serum biochemistry results were within normal limits. Urinalysis revealed a hypersthenuric urine specific gravity (1.054; reference interval [RI]: 1.005–1.030) and mild proteinuria (1 + = 30 mg/dl; RI: negative), with a normal urine protein-to-creatinine ratio. Abdominal ultrasound findings were unremarkable. Serology for *Toxoplasma gondii* and *Neospora caninum* was negative.

The patient was hospitalized for supportive care, including intravenous fluid therapy.

Magnetic resonance imaging (MRI) of the brain and cervical spine (to the level of the T2 vertebra) was performed on a 3.0-T scanner (MAGNETOM Skyra; Siemens Medical Solutions USA, Inc., Malvern, PA). A comprehensive multiplanar protocol was used, including standard sequences such as HASTE, T2-weighted (T2w), FLAIR, DWI, SWI, and post-contrast MP-RAGE and T2-weighted fat-suppressed (T2w-FS) sequences. Full imaging parameters are detailed in Supplementary Table 1.

**Figure 1:**
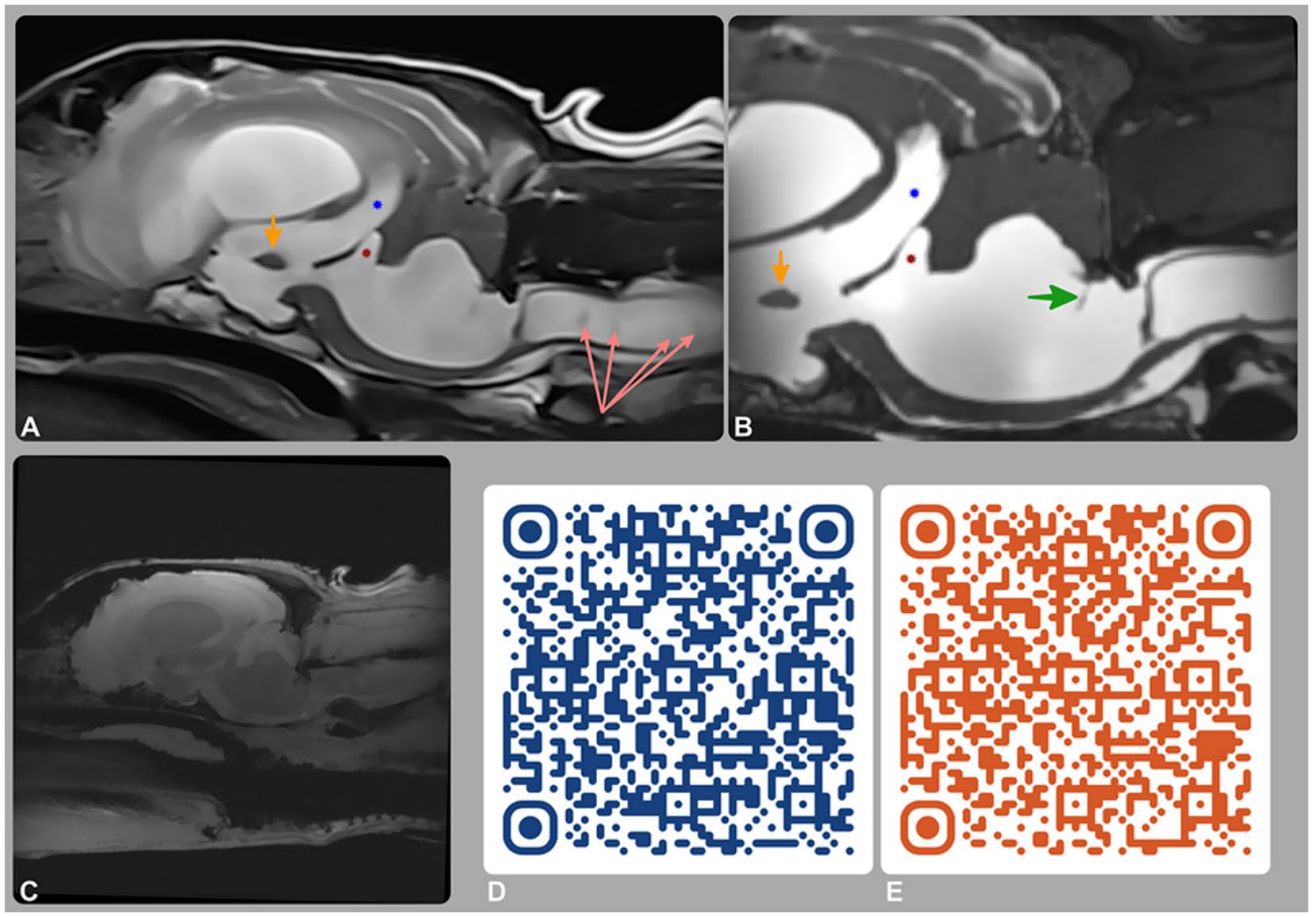
Magnetic resonance imaging (MRI) of the brain and cervical spine. **(A)** T2-weighted and **(B)** 3D-CISS mid-sagittal images; **(C–E)** phase-contrast MRI (PC-MRI), including a re-phase image **(C)** and QR-code-linked videos of the magnitude **(D)** and phase **(E)** sequences. MRI showed tetraventricular hydrocephalus, supracollicular fluid accumulation, and third (blue star) and fourth (red star) ventricle dilation. A membrane within the fourth ventricle (green arrow) represents a stretched rostral medullary velum. CSF flow was preserved intraventricularly but absent at the lateral apertures on PC-MRI, consistent with partial or complete obstruction.

**Figure 2:**
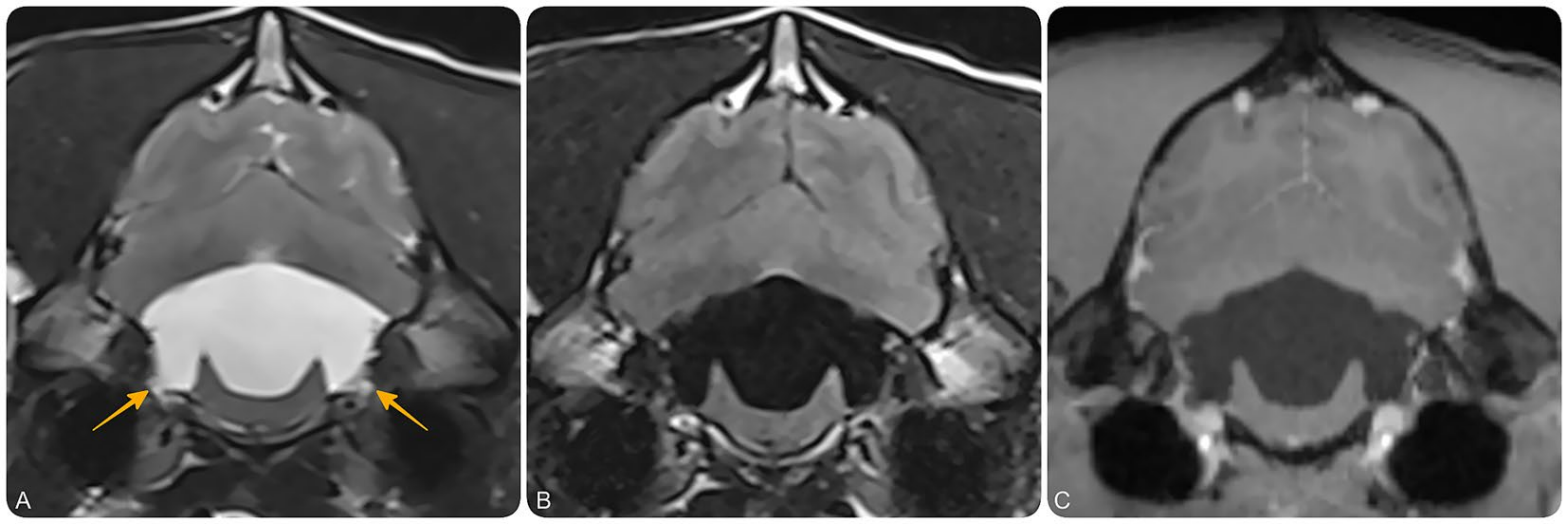
Magnetic resonance imaging (MRI) of the brain at the level of the fourth ventricle and lateral recesses. Transverse T2-weighted **(A)**, T2-FLAIR **(B)**, and T1-weighted post-contrast **(C)** MR images at the level of the lateral apertures of the fourth ventricle. These images demonstrate bilateral dilation of the lateral recesses (yellow arrows), suggestive of impaired CSF outflow at the level of the lateral apertures. No contrast-enhancing or intraventricular cystic lesions are identified.

MRI findings (Figures 1A,B) revealed severe, diffuse dilation of the entire ventricular system, including the lateral ventricles and olfactory recesses, third ventricle, mesencephalic aqueduct, fourth ventricle, and lateral recesses (Figure 2). Additional findings included flattening of the interthalamic adhesion, compression of the cerebellum and brainstem, and supracollicular fluid accumulation with concurrent expansion of both the third ventricle and the quadrigeminal cistern. Within the ventricular system and the syrinx, hyperintensity on FLAIR and mild signal voids on T2w images were consistent with rapid and turbulent CSF flow. A thin band isointense to gray matter on both T1w and T2w images was observed along the dorsal and lateral aspects of the caudal portion of the fourth ventricle. Moderate periventricular hyperintensities on T2w and FLAIR images were consistent with hydrostatic interstitial edema. Effacement of the cerebral sulci was noted, along with dorsal displacement of the corpus callosum and partial absence of the septum pellucidum. At the level of the cervical spine, moderate scoliosis and severe syringomyelia were identified, accompanied by extensive spinal cord edema extending throughout the cervicothoracic region. No abnormal parenchymal or meningeal enhancement was observed following contrast administration. The ventricle-to-brain (VB) index, assessed on dorsal T2w images, was 0.69 (reference interval < 0.6) (14).

The imaging findings were consistent with severe obstructive hydrocephalus and syringomyelia. CSF flow obstruction was suspected to result from either an arachnoid diverticulum at the fourth ventricle or an obstruction at the lateral aperture. CSF analysis from both cisternal and lumbar punctures revealed normal findings. An acquired etiology was considered most likely based on the acute clinical presentation, normal skull morphology, and imaging features indicative of increased intracranial pressure and altered CSF dynamics. While an active inflammatory process was not identified at the time of diagnosis, prior inflammation resulting in chronic membranous obstruction of the lateral apertures (e.g., post-choroiditis) is a recognized mechanism in similar cases (24, 25). A congenital etiology could not be definitively excluded but was considered less likely given the absence of typical phenotypic features and the acute onset of clinical signs.

Dexamethasone sodium phosphate (0.14 mg/kg IV q12h for 3 days, then q24h; Dexamethasone Injection, 2 mg/mL, VetOne, Boise, ID, USA) was administered, with subsequent improvement to weak ambulation within 48 h. The patient was discharged 5 days later with prednisone (1 mg/kg PO q24h; PredniSONE Tablets USP, Strides Pharma Inc., East Brunswick, NJ, USA) and omeprazole (1 mg/kg PO q12h; Omeprazole Capsules, Lek Pharmaceuticals, Ljubljana, Slovenia).

The dog was re-evaluated 12 days later (Table 1) and demonstrated marked improvement; he was active, playful, and eating well. Neurological examination revealed ongoing mild ambulatory tetraparesis and a persistent right-sided head tilt, with no evidence of ataxia. As the patient was clinically stable and uncertainty remained regarding the exact cause of hydrocephalus—whether lateral aperture obstruction or fourth ventricle arachnoid diverticulum—medical management was continued. Definitive surgical planning was postponed pending advanced MRI sequences to guide appropriate intervention, alongside logistical and scheduling constraints.

Seven weeks later, at 7 months and 2 weeks of age, the dog’s neurological condition progressively deteriorated over a two-week period, despite continued treatment with the same medications. He became non-ambulatory and developed a more pronounced right head tilt, marked shoulder muscle atrophy, and severe, diffuse tremors, which were most prominent in the thoracic limbs and eyelids (Supplementary Video 1).

As his weight increased from 17 to 25 kg, the initially prescribed doses of prednisone and omeprazole effectively decreased to 0.6 mg/kg once daily and twice daily, respectively. Consequently, both medications were adjusted to 1 mg/kg per dose.

During this time, he also developed mild superficial exfoliative pyoderma and furunculosis. A skin swab culture yielded *Staphylococcus pseudintermedius*, and a diagnosis of atopic dermatitis with secondary bacterial pyoderma was suspected. Clindamycin (12 mg/kg PO q12h for 4 weeks; Clindamycin Hydrochloride Capsules USP, Cronus Pharma, East Brunswick, NJ, USA) was initiated.

Two weeks later, at 8 months of age, the dog was re-examined and remained non-ambulatory, with no improvement in neurological status since the previous assessment. However, pressure sores had developed over the left elbow. Given the progression of clinical signs and the lack of response to medical management, surgical intervention was discussed and recommended following resolution of the skin infection.

Ten days thereafter, he was admitted for further investigations. Repeat hematology and serum biochemistry were performed and yielded unremarkable results.

A repeat brain MRI (Figures 1C–E) was performed to assess disease progression and assist with surgical planning. Advanced imaging sequences included 3D-Constructive Interference in Steady State (3D-CISS) and Phase-Contrast MRI (PC-MRI). Full acquisition parameters, including flip angle, voxel size, field of view, and gating strategy, are reported in Supplementary Table 1. These sequences provided high-resolution anatomical detail and enabled CSF flow evaluation using re-phase, magnitude, and phase images.

MRI findings revealed progressive loss of parenchymal volume, with an increase in VB index to 0.82 (RI < 0.6). A thin membrane within the fourth ventricle, better visualized on 3D-CISS, corresponded to the rostral medullary velum—a normal anatomical structure that becomes more conspicuous with ventricular dilation and altered anatomy, rather than a pathological septation or diverticulum. PC-MRI, together with conventional sequences and anatomical landmarks, demonstrated preserved intraventricular CSF flow but no signal to suggest outward flow from the region of the lateral apertures, supporting this location as the site of obstruction. These findings excluded a fourth ventricle arachnoid diverticulum and supported a diagnosis of obstructive hydrocephalus, most likely secondary to lateral aperture occlusion.

**Figure 3:**
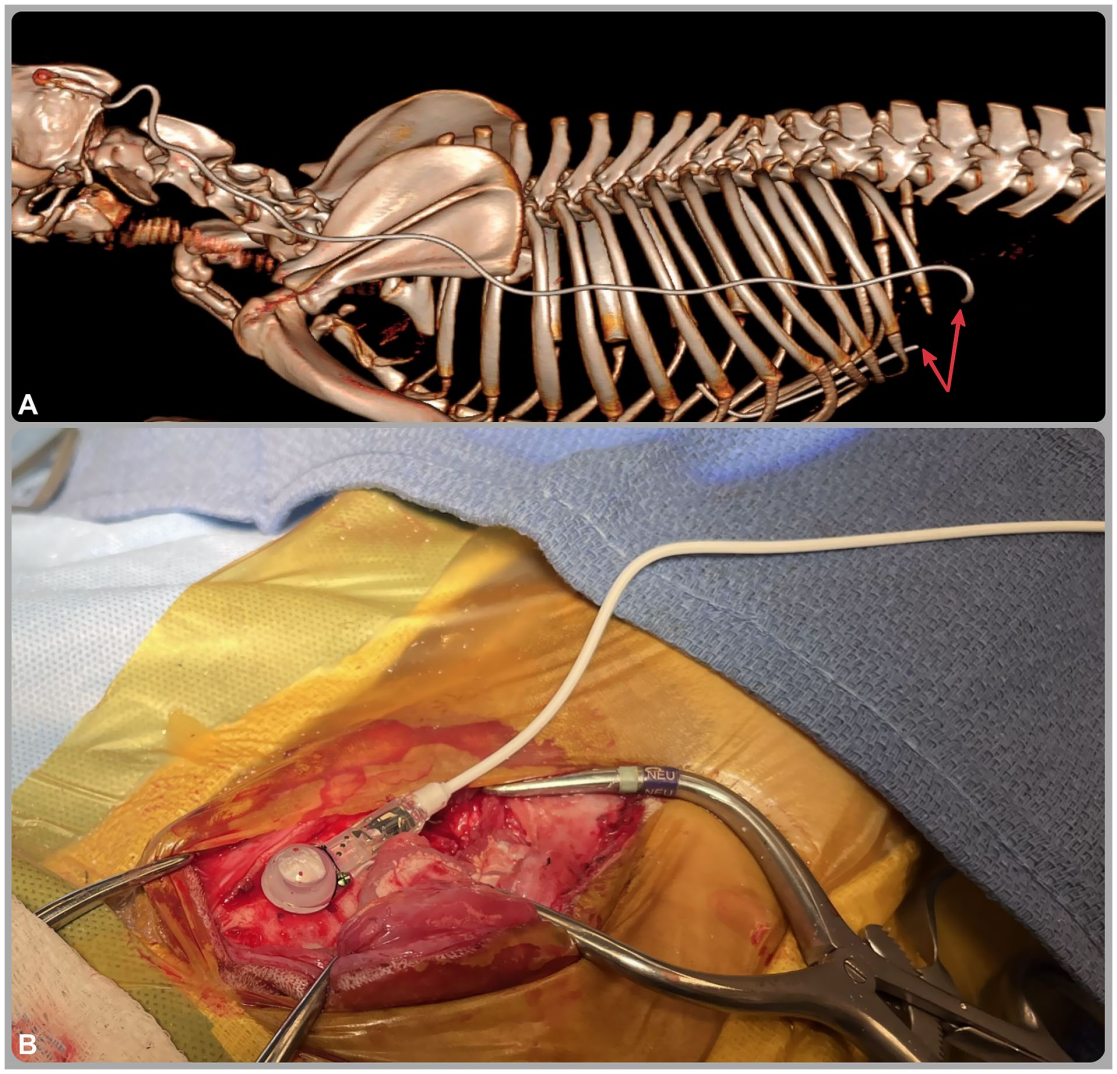
**(A)** Three-dimensional reconstructed computed tomography (CT) image from the caudal aspect of the head to the cranial lumbar spine; **(B)** intraoperative image of the ventriculoperitoneal shunt. The CT image **(A)** reveals fracture of the distal catheter (red arrows) at the level of the abdominal wall, likely secondary to patient growth. The intraoperative image **(B)** shows a Codman Hakim® right-angle medium-pressure valve (70 mm H₂O) implanted in the skull and connected to the distal catheter.

One day following imaging—and 3 months after the onset of clinical signs—a VP shunt was placed as described by Biel (12) and Schmidt (26), diverting CSF from the left lateral ventricle to the peritoneal cavity. To accommodate anticipated growth and reduce the risk of catheter migration, a redundant length of distal catheter was provided and the catheter was anchored to the abdominal wall using a Chinese finger-trap suture pattern (27). A Codman Hakim® right-angle medium-pressure valve (70 mm H₂O) was used (Figure 3B), and postoperative radiographs confirmed appropriate shunt placement.

Over the following 3 days, mild improvement was noted in the head tilt and generalized tremors (Supplementary Video 2). The dog was discharged 1 week later with instructions to continue clindamycin (12 mg/kg PO q12h for 6 weeks; Clindamycin Hydrochloride Capsules USP, Cronus Pharma, East Brunswick, NJ, USA), diazepam (0.2 mg/kg PO q8h PRN; Diazepam Tablets USP, Teva Pharmaceuticals, Parsippany, NJ, USA), gabapentin (12 mg/kg PO q8–12h PRN; compounded Gabapentin Capsules, NC State University, Raleigh, NC, USA), prednisone (1 mg/kg PO q24h for 1 week, tapered to 0.4 mg/kg q24h for 4 weeks, 0.2 mg/kg q24h for 2 weeks, and 0.1 mg/kg q48h for 10 days before discontinuation; PredniSONE Tablets USP, Strides Pharma Inc., East Brunswick, NJ, USA), and omeprazole (1 mg/kg PO q12h; Omeprazole Capsules, Lek Pharmaceuticals, Ljubljana, Slovenia).

One week after VP shunt placement, the dog regained the ability to ambulate independently. Eighty days post-surgery, at 11 months of age, the dog was re-evaluated and showed strong ambulation, with only moderate thoracic limb hypermetria as a residual neurological deficit. At that time, he was still receiving omeprazole at the same dosage, which had been continued at home following discharge. Given the sustained clinical improvement, omeprazole was subsequently discontinued.

Five months after surgery, at 13 months of age, the dog experienced acute neurological deterioration, developing non-ambulatory tetraparesis with tremors primarily affecting the thoracic limbs and face—similar to the clinical presentation prior to VP shunting. Mild discomfort was also elicited on cervical spinal palpation, a finding not noted at the initial presentation.

A CT scan was performed to assess shunt integrity and positioning. Findings indicated undershunting due to fracture of the distal catheter at its anchoring site in the abdominal wall (Figure 3A). Despite the extra catheter length, the patient’s weight increase from 25 to 42 kg, combined with firm fixation to both the abdominal musculature and parietal peritoneum (12, 26, 27), likely resulted in mechanical strain, leading to catheter fracture. During revision surgery, the fractured distal catheter was trimmed and extended with a titanium connector, then re-anchored solely to the abdominal musculature to reduce mechanical stress and avoid recurrence. Shunt patency was confirmed intraoperatively, with CSF observed flowing from the proximal end of the distal catheter at the rupture site.

Postoperatively, a radiograph confirmed correct shunt positioning. Within 24 h, the patient showed substantial neurological improvement, regaining ambulation, although severe cerebellar tetra-ataxia persisted. He was discharged 2 days later with gabapentin (10 mg/kg PO q8–12h PRN; compounded Gabapentin Capsules, NC State University, Raleigh, NC, USA), prednisone (0.25 mg/kg PO q24h for 3 days, then tapered to 0.1 mg/kg q48h for three doses before discontinuation; PredniSONE Tablets USP, Strides Pharma Inc., East Brunswick, NJ, USA), and cefpodoxime proxetil (7 mg/kg PO q12h for 1 week; Cefpoderm, Dechra Pharmaceuticals, Fort Worth, TX, USA).

Seven months after revision surgery, at 20 months of age, the dog was neurologically improved, with persistent, mild cerebellar tetra-ataxia primarily characterized by hypermetria in the thoracic limbs.

**Figure 4:**
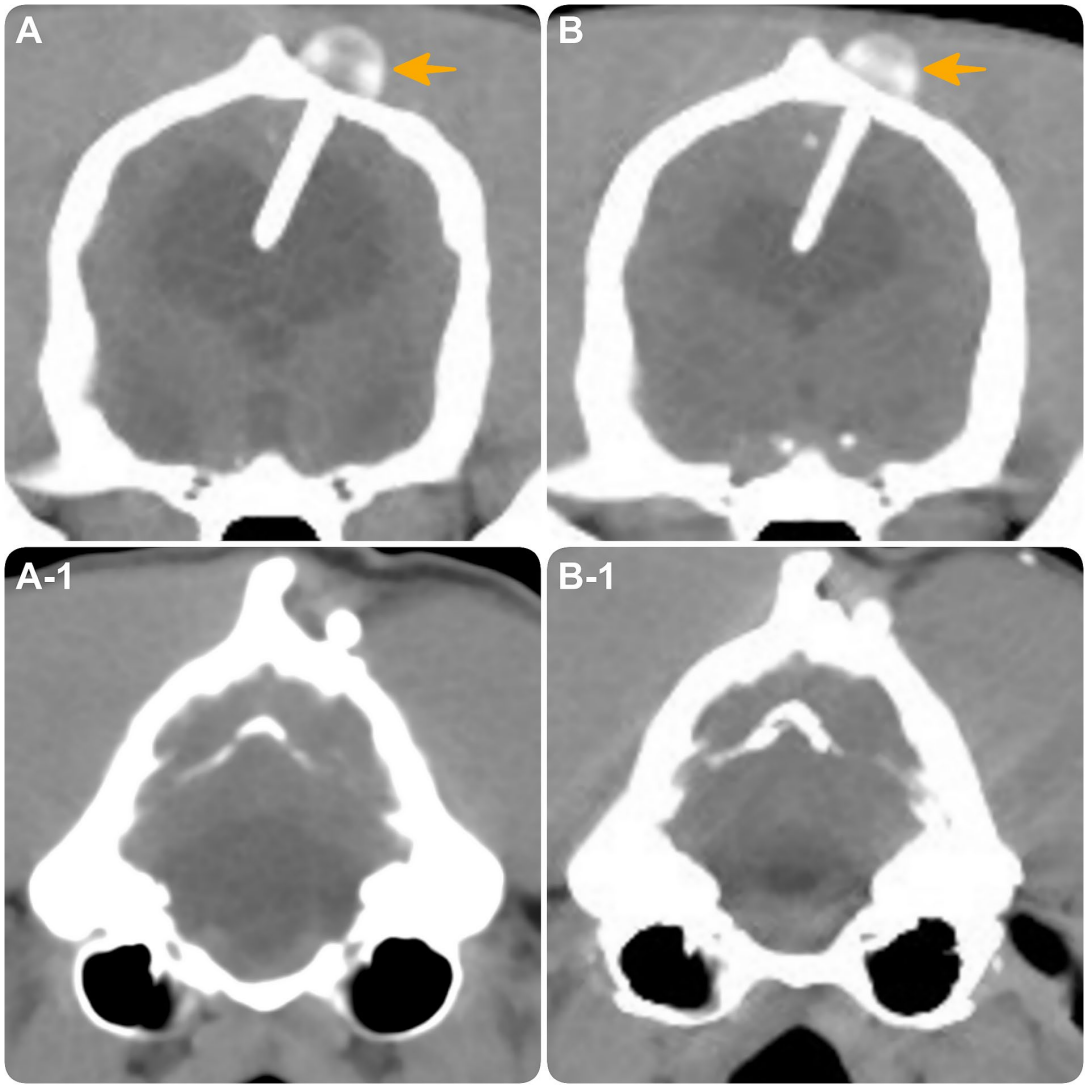
Transverse CT images at the level of the interthalamic adhesion **(A,B)** and the fourth ventricle **(A1,B1)**. **(A,A-1)** obtained at the time of distal catheter fracture. **(B,B-1)** obtained 10 months post-revision surgery, demonstrating intact shunt positioning (yellow arrow) with moderate reduction in hydrocephalus and expansion of brain parenchyma.

Three and a half months later, at 24 months of age and 10 months post-revision surgery, the dog was clinically unchanged (Supplementary Video 3). Repeat CT scan revealed no concerns with the VP shunt and demonstrated moderate reduction in hydrocephalus with noticeable brain parenchyma expansion compared to the CT at the time of catheter fracture (Figure 4).

At the most recent in-person evaluation, at 28 months of age and 15 months post-revision surgery, the dog remained clinically stable.

## 3 Discussion

This case report describes a dog with non-communicating hydrocephalus, most likely acquired given the acute onset, normal skull morphology, and MRI findings consistent with increased intracranial pressure and altered CSF dynamics. Despite normal CSF analysis, prior inflammation causing chronic membranous obstruction of the lateral apertures was considered the most plausible mechanism (13, 24), although this could not be confirmed. While the initial conventional MRI findings, interpreted in light of previously published reports (28), raised the possibility of a fourth ventricle arachnoid diverticulum, advanced sequences (3D-CISS and PC-MRI) excluded this and confirmed obstruction at the level of the lateral apertures. Misinterpretation in similar cases could lead to an inappropriate surgical approach—such as suboccipital craniectomy for diverticulum removal—which has been associated with poor outcomes (28).

Although the advanced MRI sequences in this case were instrumental in confirming the diagnosis and guiding the correct surgical intervention (VP shunting), in many instances the diagnosis of lateral aperture obstruction can be made from conventional MRI sequences alone. Findings such as tetraventricular hydrocephalus, enlargement of the lateral recesses, and absence of an alternative obstructive lesion support this diagnosis. As these advanced sequences require specific software and hardware and may not be feasible in all institutions—particularly with low-field or non-cardiac gated systems—their absence should not delay urgent surgical intervention when the obstruction is otherwise evident.

In retrospect, it is possible that some previously published cases with similar MRI findings (28) represented lateral aperture obstruction rather than fourth ventricle arachnoid diverticula. While those interpretations reflected the understanding at the time, greater awareness of the characteristic MRI features—such as those seen in the present case— may help reduce diagnostic uncertainty and aid in selecting the most appropriate surgical approach.

PC MRI relies on bipolar gradients, which are pairs of gradients with equal magnitude and duration but opposite polarity (29). These gradients are applied after the radiofrequency pulse and before signal acquisition during the echo time (TE) and allow the detection of phase shifts caused by fluid motion (29). Data acquisition is synchronized with the cardiac cycle using electrocardiographic (ECG) gating, as in the present case, or alternatively by pulse oximetry or plethysmography (29). Qualitative assessment of CSF flow direction and dynamics was also performed in this case using images acquired in the sagittal plane (29). Quantitative analysis of CSF velocity and flow characteristics is also possible when images are acquired in a through-plane orientation, perpendicular to the direction of flow (29). In such cases, key anatomical constriction points such as the mesencephalic aqueduct or foramen magnum are typically selected for imaging (29). However, this quantitative evaluation requires dedicated post-processing software and was not performed in the present case (29).

This technique yields three types of images: re-phase, magnitude, and phase images (29). The re-phase image is a flow-compensated magnitude image in which motion effects are canceled (29). It provides an anatomical overview with CSF appearing as a bright signal, though it does not convey information about flow direction or velocity (29). The magnitude image shows the absolute value of phase differences, highlighting flowing CSF as bright against a black background of stationary tissues (29). Like the re-phase image, it lacks directional or velocity information (29). The phase image, by contrast, captures true directional flow by detecting phase shifts using bipolar gradients (29). During systole, the influx of arterial blood causes slight expansion of the brain parenchyma, leading to an increase in intracranial pressure and resulting in caudal displacement of CSF through the foramen magnum into the spinal subarachnoid space (29). This rostral-to-caudal flow appears bright, corresponding to positive phase (29). During diastole, as cerebral blood volume decreases, pressure changes induce caudal-to-rostral flow, corresponding to a negative phase shift, against a mid-gray background (29). These sequences enable detailed qualitative and quantitative analysis of CSF flow direction, velocity, and volume (29).

In the case described, PC-MRI confirmed patency of intraventricular CSF flow and suggested impaired flow at the level of the lateral apertures (Figures 1C–E). Although a CSF velocity study using a quantitative flow analysis at the lateral apertures might have provided more definitive insight, this was not possible due to the lack of the post-processing software.

The 3D-CISS sequence is known for its high-resolution imaging of CSF spaces and surrounding anatomical structures (30, 31). The dorsal surface of the pons and medulla is normally covered from rostral to caudal by the rostral medullary velum, the cerebellum, and the caudal medullary velum (8). In this dog, a thin membrane within the fourth ventricle was most likely part of these normal structures, which dorsally bound the ventricle and attach to the cerebellum and its meningeal coverings. With severe ventricular dilation, these membranes became more conspicuous on MRI due to displacement and CSF accumulation, rather than representing a new or abnormal septating membrane (Figure 1B). This interpretation was supported by the complementary findings on PC-MRI.

Ventriculography remains the definitive method to confirm obstruction of CSF outflow; however, its invasiveness and associated risks preclude routine use (24, 32, 33). Accordingly, advanced MRI techniques, as applied here, offer a safer alternative for etiological assessment and surgical planning (24, 33). In this case, PC-MRI and 3D-CISS sequences supported a diagnosis of obstructive hydrocephalus, most likely due to lateral aperture occlusion, guiding the decision to place a VP shunt.

As previously reported by several authors (12, 15–22), medical therapy for hydrocephalus in dogs—most commonly involving corticosteroids, acetazolamide, omeprazole, or furosemide—may offer only transient clinical improvement by reducing CSF production or periventricular interstitial edema. However, these treatments do not address the underlying cause of CSF accumulation. Consequently, neurological deterioration is common, and long-term stability usually requires surgical intervention. In our case, the patient remained neurologically stable at 23 months after the initial presentation—including 15 months post-revision—supporting the long-term efficacy of the selected surgical approach, with only mild-to-moderate residual cerebellar tetra-ataxia.

Wyatt (34) described a case in which a transcerebellar VP shunt was placed after a lateral ventricular shunt had resolved supratentorial hydrocephalus but failed to decompress the fourth ventricle. This was interpreted at the time as a fourth ventricle arachnoid diverticulum; however, retrospective review suggests that some MRI features—particularly dilation of the lateral recesses—could also be consistent with lateral aperture obstruction. In such scenarios, differentiating between these entities is important, as the optimal surgical approach may differ. When advanced MRI sequences are unavailable, a stepwise strategy may be reasonable—initially addressing tetraventricular hydrocephalus with a lateral VP shunt, with consideration of a transcerebellar shunt only if persistent fourth ventricle dilatation is confirmed and deemed clinically significant.

Postoperative complications following VP shunt placement in dogs typically occur within the first 3 months, with reported incidence rates ranging from 22 to 29% (1, 12, 15, 26, 34–38). The most common complications include shunt malfunction—most often due to obstruction (1, 12, 15, 26, 34, 37)—and infection (12, 15, 37, 38). Obstruction may arise from intraluminal blockage, kinking, coiling, migration, or disconnection of the catheter, while infection is most commonly associated with skin flora such as *Staphylococcus* spp. Seizures may persist, resolve, or develop after surgery; when new seizures occur, it is often unclear whether they represent a true shunt-related complication or progression of the underlying disease (15, 35, 36).

In our case, shunt-related complications emerged 5 months postoperatively—later than typically reported—due to undershunting caused by mechanical fracture of the distal catheter at its anchoring site. Although additional catheter length had been left to accommodate growth, the dog’s substantial weight gain (from 25 to 42 kg) may have imposed excessive tensile stress on the system, exacerbated by firm fixation to both the abdominal musculature and parietal peritoneum using a Chinese finger-trap suture. While this technique helps prevent catheter migration (27), we hypothesize that, in this instance, it may have contributed to mechanical fatigue and eventual failure.

At revision, the catheter was reattached solely to the abdominal musculature using the same suture technique and intentionally left with excess intraperitoneal length. This strategy was intended to reduce mechanical stress during movement and ongoing development. In growing large-breed dogs, avoiding any rigid fixation altogether—allowing the catheter to move more freely within the peritoneal cavity, supported instead by subcutaneous tunneling, layered closure, and redundant intraperitoneal length—may represent a viable alternative. However, further investigation is warranted to determine whether such approaches mitigate the risk of growth- or motion-related mechanical failure.

As hydrocephalus more commonly affects small and toy breeds (1, 2), this type of complication is rarely encountered, highlighting the importance of accounting for anticipated growth when placing VP shunts in larger or immature patients.

Shunt revision has been shown to resolve postoperative complications in over 70% of cases (12). Consistent with these findings, our patient experienced rapid and marked clinical improvement following surgical revision, returning to their pre-complication neurological status, with only mild residual deficits remaining.

Selecting an appropriate pressure valve for VP shunting can be challenging, as overdrainage may result in serious complications, including collapse of the lateral ventricles (3, 39). Some studies suggest that mild undershunting may still yield clinical improvement (23, 40), making it a potentially safer initial strategy. In this case, a medium-pressure valve was selected, resulting in reduced ventricular volume and expansion of brain parenchyma postoperatively. This was associated with a marked improvement in the patient’s clinical status.

Despite the use of advanced MRI sequences, certain limitations remain. Quantitative CSF velocity analysis at the level of the lateral apertures was not performed due to the lack of dedicated post-processing software, limiting definitive confirmation of the source of CSF flow obstruction. Additionally, ventriculography—which could provide direct visualization of CSF outflow and confirm lateral aperture obstruction—was not pursued due to its invasiveness and associated risks.

Postoperative imaging was limited to CT, which confirmed appropriate shunt placement and a moderate reduction in ventricular size. However, MRI follow-up would have provided a more detailed evaluation of both brain and cervical spinal parenchymal changes and postoperative CSF dynamics. In addition, CSF flow was not assessed in the cervical spinal region, limiting insight into potential flow-related alterations beyond the brain.

While the diagnosis of lateral aperture obstruction remains presumptive without histopathological confirmation, the combination of characteristic imaging findings—including tetraventricular hydrocephalus, dilated lateral recesses, and the absence of alternative obstructive lesions—strongly supports this diagnosis. These features, in conjunction with anatomical knowledge and published reference data (24) may be sufficient to guide clinical decision-making in many cases, particularly when advanced imaging techniques are unavailable.

## 4 Conclusion

We describe a case of severe hydrocephalus with marked syringomyelia in a young dog, likely due to lateral aperture obstruction. Advanced MRI sequences—particularly 3D-CISS and PC-MRI—were instrumental in supporting the diagnosis, excluding fourth ventricle arachnoid diverticula, and enabling a tailored surgical approach. While conventional MRI may suffice in many cases, advanced imaging improves diagnostic confidence and surgical planning.

VP shunting was selected over suboccipital craniectomy based on imaging findings. In large, growing dogs, VP shunt placement should account for somatic growth; avoiding rigid fixation and ensuring intraperitoneal mobility and subcutaneous redundancy may reduce the risk of mechanical catheter failure.

## Data Availability

The raw data supporting the conclusions of this article will be made available by the authors, without undue reservation.

## Supplementary material

SUPPLEMENTARY VIDEO 1 Clinical video showing the dog’s non-ambulatory status, right head tilt, cervical scoliosis/torticollis, and severe, diffuse tremors—most prominent in the thoracic limbs and eyelids—at the time of presentation.

SUPPLEMENTARY VIDEO 2 Clinical video showing mild improvement in the dog’s right head tilt and generalized tremors following placement of the ventriculoperitoneal shunt.

SUPPLEMENTARY VIDEO 3 Clinical video showing the dog at the time of the last follow-up.

SUPPLEMENTARY TABLE 1 MRI protocol detailing conventional sequences and advanced imaging for CSF flow assessment, including 3D-CISS and PC-MRI.
