# Supplementary material for: Case Report: 3D-CISS and PC-MRI in the diagnosis and surgical planning of hydrocephalus secondary to presumptive lateral aperture obstruction in a dog
Source: Front Vet Sci. 2025 Oct 10;12:1646137. doi: 10.3389/fvets.2025.1646137 (PMC12551229; doi:10.3389/fvets.2025.1646137)
Supplement: SUPPLEMENTARY TABLE 1 — MRI protocol detailing conventional sequences and advanced imaging for CSF flow assessment, including 3D-CISS and PC-MRI. [file Supplementary_Table_1.DOCX]

# **Supplementary Table 1.** MRI protocol detailing conventional sequences and advanced imaging for CSF flow assessment, including 3D-CISS and PC-MRI.

## First MRI Protocol:

| Sequence Type | Plane | TE (ms) | TR (ms) | Slice Thickness |
| --- | --- | --- | --- | --- |
| HASTE | Sagittal | 997 | 2580 | 3 mm |
| HASTE | Dorsal | 246 | 2010 | 3 mm |
| T2-weighted | Sagittal | 98 | 1120 | 3 mm |
| T2-weighted | Transverse | 100 | 2900 | 2 mm |
| T2-weighted | Dorsal | 98 | 1500 | 2 mm |
| FLAIR | Transverse | 91 | 9000 | 2 mm |
| DWI | Transverse | 70 | 6800 | 3 mm |
| SWI | Transverse | 20.9 | 43 | 1.5 mm |
| MP-RAGE (pre- and post-contrast) | Dorsal (reformatted sagittal/transverse/dorsal) | 3.7 | 2310 | 0.7 mm |
| T2-weighted Fat-Suppressed (post-contrast) | Sagittal | 112 | 2500 | 3 mm |
| T2-weighted Fat-Suppressed (post-contrast) | Dorsal | 113 | 2000 | 3 mm |

**Second MRI Protocol (Advanced Sequences):**

| Sequence Type | Plane | TE (ms) | TR (ms) | Slice Thickness | Notes |
| --- | --- | --- | --- | --- | --- |
| 3D-CISS | Sagittal | 3.77 | 8.32 | 0.6 mm | High-resolution anatomic detail |
| PC-MRI (Phase Contrast) | Sagittal | 7.78 | 23.68 | 6 mm (single slice) | Re-phase, magnitude, and phase images acquired for CSF flow assessment |

**Second MRI Protocol (Advanced Sequences – Full Parameter Set):**

| Sequence Type | Parameters |
| --- | --- |
| 3D-CISS | Plane: Sagittal TE/TR: 3.77/8.32 ms Slice thickness: 0.6 mm Flip angle: 50° Bandwidth: 460 Hz/pixel FOV: 86 × 139 mm Matrix: 512 × 318 Voxel size: 0.27 × 0.27 × 0.50 mm Averages: 1 Notes: High-resolution anatomic detail |
| PC-MRI (Phase Contrast) | Plane: Sagittal TE/TR: 7.78/23.68 ms Slice thickness: 6 mm (single slice) Flip angle: 10° FOV: 180 × 180 mm Matrix: 256 × 205 (pixel spacing 0.703 × 0.703 mm) Bandwidth: 201 Hz/pixel Averages: 1 Retrospective ECG gating used (40 cardiac phases, nominal RR: 1326 ms, ~180 heartbeats recorded, trigger delay ~331.5 ms) Velocity encoding: In-plane (foot-to-head) Notes: Re-phase, magnitude, and phase images acquired for CSF flow assessment |
